# SARS-CoV-2 spike protein: a key target for eliciting persistent neutralizing antibodies

**DOI:** 10.1038/s41392-021-00523-5

**Published:** 2021-02-26

**Authors:** Yang Yang, Lanying Du

**Affiliations:** 1 Roy J. Carver Department of Biochemistry, Biophysics and Molecular Biology, Iowa State University, Ames, IA USA; 2 Howard Hughes Medical Institute, Yale University, New Haven, CT USA; 3 Department of Molecular, Cellular and Developmental Biology, Yale University, New Haven, CT USA; 4 Lindsley F. Kimball Research Institute, New York Blood Center, New York, NY USA

**Keywords:** Microbiology, Infectious diseases

A recent paper published in *Science* describes the detection of IgG antibody responses in individuals infected by severe acute respiratory syndrome coronavirus (SARS-CoV-2). The authors also examined the duration of antibody production and the correlation between IgG antibody titers and neutralizing antibody titers.^1^ This study provides information about the kinetics of antibody production, and the functionality and longevity of these antibodies, in patients with Coronavirus Disease 2019 (COVID-19).

**Fig. 1:**
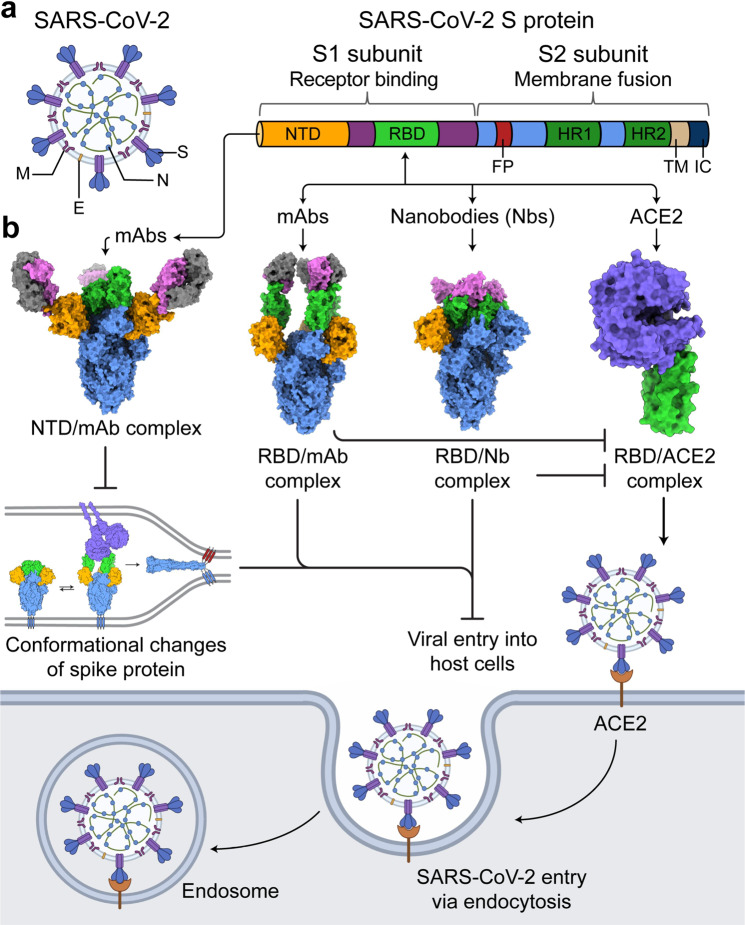
SARS-CoV-2 spike (S) protein is a key target for eliciting neutralizing antibodies. **a** Schematic structures of SARS-CoV-2 virion and its S protein. M, membrane; E, envelope; N, nucleocapsid. Viral RNA is located inside the virion. NTD, N-terminal domain; RBD, receptor-binding domain; FP, fusion peptide; HR1 and HR2, heptad region 1 and 2; TM, transmembrane domain; IC, intracellular tail. **b** Mode of action of SARS-CoV-2 S-specific neutralizing antibodies. Monoclonal antibodies (mAbs) targeting S protein NTD prevent conformational changes of the S protein that are required for S2-mediated membrane fusion, and hence inhibit viral entry into host cells. RBD-targeting neutralizing mAbs or nanobodies (Nbs), on the other hand, bind directly to SARS-CoV-2 S protein RBD and compete with the cellular receptor, angiotensin converting enzyme 2 (ACE2), resulting in neutralization of viral infection and clearance of the virus. The following PDB entries are used for structural illustrations: 7C2L (structure of SARS-CoV-2 S in complex with NTD-targeting mAb 4A8), 7K4N (structure of SARS-CoV-2 S in complex with RBD-targeting mAb S2E12), 7KKK (structure of SARS-CoV-2 S in complex with RBD-targeting nanobody Nb6), and 6VW1 (structure of SARS-CoV-2 RBD in complex with human ACE2). SARS-CoV-2 S NTD is colored in orange, RBD in green, and the rest part of S protein in light blue. ACE2 is colored in purple. This figure was prepared using BioRender (https://biorender.com/) and UCSF ChimeraX (https://www.cgl.ucsf.edu/chimerax/)

The SARS-CoV-2 genome encodes spike (S), nucleocapsid, membrane, and envelope structural proteins. The S protein plays a key role in viral infection and pathogenesis.^2^ It comprises subunits S1 and S2: S1 harbors the N-terminal domain (NTD) and the receptor-binding domain (RBD), whereas S2 harbors heptad repeat 1 (HR1) and HR2 (Fig. 1a). SARS-CoV-2 infection undergoes a series of processes: the RBD first binds its receptor, angiotensin converting enzyme 2 (ACE2), to form an RBD/ACE2 complex. This triggers conformational changes in the S protein, leading to membrane fusion mediated via HR1 and HR2; this process culminates in viral entry into target cells (Fig. 1b). Different from other structural proteins, the S protein is a critical target for the induction of antibodies, particularly neutralizing antibodies, specific for SARS-CoV-2. Antibodies targeting various regions of S protein have different mechanisms in inhibiting SARS-CoV-2 infection. For example, NTD-targeting antibodies (monoclonal antibodies (mAbs) or their fragments) bind the NTD to form an NTD/mAb complex, thereby preventing conformational changes in the S protein and blocking membrane fusion and viral entry (Fig. 1b). By contrast, RBD-targeting antibodies such as mAbs and nanobodies (Nbs) form RBD/mAb or RBD/Nb complexes that inhibit binding of the RBD to ACE2, thereby preventing entry of SARS-CoV-2 into target cells (Fig. 1b). Thus, understanding the aforementioned mechanism underlying SARS-CoV-2 infection and the mode of action of anti-SARS-CoV-2-S antibodies will help elucidate the kinetics of antibody production in SARS-CoV-2-infected individuals, and facilitate the development of effective countermeasures. In general, antibodies targeting the viral RBD are more potent than the antibodies targeting other regions (such as NTD) of S protein, but they might be less broad in inhibiting multiple virus strains.

The *Science* paper shows that anti-SARS-CoV-2-S antibodies are elicited at detectable titers after infection.^1^ The authors detected IgG antibodies in convalescent plasma from patients with mild-to-moderate COVID-19 symptoms. They performed an ELISA by coating plates with a stabilized SARS-CoV-2 S trimer protein. Screening 72,401 samples yielded a positive rate of 41.5% (30,082/72,201), with IgG titers of ≥1:80; the majority of positive samples contained moderate (1:320 or 1:960) to high (1:2880) titers of S-specific antibodies.

They then selected 120 plasma samples with IgG titers varying from non-detectable to ≥1:2880 and examined neutralizing activity against authentic SARS-CoV-2 infection. Plasma with IgG titers of 1:320, 1:960, or ≥1:2880 corresponded to neutralizing antibody titers of 1:30, 1:75, and 1:550, respectively, and all samples with IgG titers ranging from 1:960 to ≥1:2880 neutralized SARS-CoV-2 infection. Overall, these results indicate that SARS-CoV-2 S-targeting IgG antibodies in SARS-CoV-2-infected patients neutralized authentic SARS-CoV-2 infection, and that the induced IgG antibody titer correlated with the neutralizing antibody titer. The data from this study are consistent with those from previous studies on SARS-CoV and Middle East respiratory syndrome coronavirus (MERS-CoV), other coronaviruses with high fatality rates in humans; the latter studies show that, virus-specific antibodies, particularly those targeting S proteins and/or RBD fragments, correlate positively with neutralizing antibody titers.^2^ In addition, the *Science* paper also examined longevity of IgG antibodies specific for the SARS-CoV-2 S protein in 121 volunteers over a period of around 5 months, separated by three mean time points (30, 82, and 148 days after symptom onset).^1^ Notably, specific antibodies were maintained for at least 5 months, showing a modest decrease over time (particularly at initial titers of 1:960 to ≥1:2880). Moreover, neutralizing antibody titers still correlated with neutralizing antibody titers at Day 148. As pointed out by the authors, detection and measurement of antibodies generated by SARS-CoV-2 infection should be extended over a longer period of time. This may give indications as to whether people infected with SARS-CoV-2 can be reinfected and, if so, at which antibody level. In the case of SARS-CoV and MERS-CoV, high-titer S/RBD-specific IgG and neutralizing antibodies can be maintained for up to 12 months post-vaccination, providing complete protection against viral infection.^2^ It is therefore expected that the higher the titer of specific IgG antibodies with neutralizing activity against SARS-CoV-2 infection, and the longer they persist, the more effective they will be in preventing reinfection.

IgG antibodies induced by SARS-CoV-2 in patients with moderate-to-severe symptoms mainly target the S protein;^1^ however, it is unclear which regions (RBD, NTD, or other fragments) of the S protein are targeted, or whether the induced antibodies show cross-reactivity and/or cross-neutralizing activity against other coronaviruses. Some studies indicate that SARS-CoV-2 RBD-specific IgG antibodies are present in convalescent plasma from COVID-19 patients, and that IgG antibodies specific for RBD and S (ectodomain) correlate positively with anti-SARS-CoV-2 neutralizing antibody titers.^3^ Other studies demonstrate the generation of persistent, but slightly reduced, IgG antibodies in patients with COVID-19; these antibodies also target SARS-CoV-2 RBD, and the titers correlate with S-specific neutralizing antibody titers.^4^ Moreover, these antibodies may cross-react with the S/RBD of SARS-CoV, thereby cross-neutralizing SARS-CoV infection; however, they show low to no cross-reactivity with low-pathogenic human coronaviruses such as HKU1, 229E, OC43, or NL63.^4,5^ Considering the high sequence similarity between SARS-CoV-2, SARS-CoV, and other SARS-like coronaviruses, and their use of the same ACE2 receptor, further studies are needed to understand whether antibodies generated by SARS-CoV-2-infected patients cross-react with S proteins or their fragments (S1-RBD/S2), and/or cross-neutralize infections by SARS-like coronaviruses with pandemic potential.

Studies of highly pathogenic human coronaviruses (SARS-CoV-2, SARS-CoV, and MERS-CoV) indicate that, although full-length and fragments (NTD and RBD) of S proteins induce specific antibodies with neutralizing activity, the RBD is a major target for eliciting highly potent neutralizing antibodies with protective efficacy.^2^ The majority of the currently developed anti-SARS-CoV-2 therapeutic antibodies target the S protein, particularly the RBD. Therefore, the S/RBD-specific antibodies have great potential for development as effective therapeutics that prevent COVID-19 spread.
